# Effects of deep brain stimulation on quantitative sleep electroencephalogram during non-rapid eye movement in Parkinson’s disease

**DOI:** 10.3389/fnhum.2023.1269864

**Published:** 2023-09-21

**Authors:** Adeel A. Memon, Brandon S. Edney, Alexander J. Baumgartner, Alan J. Gardner, Corina Catiul, Zachary T. Irwin, Allen Joop, Svjetlana Miocinovic, Amy W. Amara

**Affiliations:** ^1^Department of Neurology, West Virginia University Rockefeller Neuroscience Institute, Morgantown, WV, United States; ^2^School of Medicine, University of Alabama at Birmingham, Birmingham, AL, United States; ^3^Department of Neurology, University of Colorado Anschutz Medical Campus, Aurora, CO, United States; ^4^Department of Neurosurgery, University of Colorado Anschutz Medical Campus, Aurora, CO, United States; ^5^Neuroscience Undergraduate Program, University of Alabama at Birmingham, Birmingham, AL, United States; ^6^Department of Neurology, University of Alabama at Birmingham, Birmingham, AL, United States; ^7^Department of Neurosurgery, University of Alabama at Birmingham, Birmingham, AL, United States; ^8^Department of Neurology, Emory University, Atlanta, GA, United States

**Keywords:** Parkinson’s disease, sleep spindles, sleep, cognition, sleep qEEG, Deep brain stimulation

## Abstract

**Introduction:**

Sleep dysfunction is frequently experienced by people with Parkinson’s disease (PD) and negatively influences quality of life. Although subthalamic nucleus (STN) deep brain stimulation (DBS) can improve sleep in PD, sleep microstructural features such as sleep spindles provide additional insights about healthy sleep. For example, sleep spindles are important for better cognitive performance and for sleep consolidation in healthy adults. We hypothesized that conventional STN DBS settings would yield a greater enhancement in spindle density compared to OFF and low frequency DBS.

**Methods:**

In a previous within-subject, cross-sectional study, we evaluated effects of low (60 Hz) and conventional high (≥130 Hz) frequency STN DBS settings on sleep macroarchitectural features in individuals with PD. In this *post hoc*, exploratory analysis, we conducted polysomnography (PSG)-derived quantitative electroencephalography (qEEG) assessments in a cohort of 15 individuals with PD who had undergone STN DBS treatment a median 13.5 months prior to study participation. Fourteen participants had unilateral DBS and 1 had bilateral DBS. During three nonconsecutive nights of PSG, the participants were assessed under three different DBS conditions: DBS OFF, DBS LOW frequency (60 Hz), and DBS HIGH frequency (≥130 Hz). The primary objective of this study was to investigate the changes in sleep spindle density across the three DBS conditions using repeated-measures analysis of variance. Additionally, we examined various secondary outcomes related to sleep qEEG features. For all participants, PSG-derived EEG data underwent meticulous manual inspection, with the exclusion of any segments affected by movement artifact. Following artifact rejection, sleep qEEG analysis was conducted on frontal and central leads. The measures included slow wave (SW) and spindle density and morphological characteristics, SW-spindle phase-amplitude coupling, and spectral power analysis during non-rapid eye movement (NREM) sleep.

**Results:**

The analysis revealed that spindle density was significantly higher in the DBS HIGH condition compared to the DBS LOW condition. Surprisingly, we found that SW amplitude during NREM was significantly higher in the DBS LOW condition compared to DBS OFF and DBS HIGH conditions. However, no significant differences were observed in the other sleep qEEG features during sleep at different DBS conditions.

**Conclusion:**

This study presents preliminary evidence suggesting that conventional HIGH frequency DBS settings enhance sleep spindle density in PD. Conversely, LOW frequency settings may have beneficial effects on increasing slow wave amplitude during sleep. These findings may inform mechanisms underlying subjective improvements in sleep quality reported in association with DBS. Moreover, this work supports the need for additional research on the influence of surgical interventions on sleep disorders, which are prevalent and debilitating non-motor symptoms in PD.

## Introduction

Although non-motor Parkinson disease (PD) features have been recognized since the original description of the disease by James Parkinson in 1817, only recently have the prevalence and impact of these non-motor symptoms become the focus of intense study (Parkinson, 2002; Chaudhuri et al., 2006). Sleep disorders are among the most common non-motor manifestations of PD, affecting 64–98% of patients (Lees et al., 1988; Barone et al., 2009). As sleep contributes to the regulation of many physiological homeostatic processes, sleep disturbance has a significant impact on quality of life in PD (Gallagher et al., 2010; Gómez-Esteban et al., 2011; Avidan et al., 2013). Though numerous symptomatic therapies exist, the treatment of sleep disorders in PD is limited by a lack of adequately powered, randomized studies providing high quality evidence (Chahine et al., 2017; Baumgartner et al., 2021).

Deep brain stimulation (DBS) is an established, effective therapy for the treatment of motor symptoms of PD (Deuschl et al., 2006; Schuepbach et al., 2013; Krack et al., 2019), though studies have shown that DBS can also improve non-motor symptoms, including sleep (Arnulf et al., 2000; Iranzo et al., 2002; Monaca et al., 2004). Subthalamic nucleus (STN) DBS has been shown to increase total sleep time, decrease wakefulness after sleep onset, and increase time spent in non-rapid eye movement (NREM) stage 2 (N2) sleep (Arnulf et al., 2000; Monaca et al., 2004). Still, results are mixed, with other studies showing a trend towards a decrease in N2 sleep (though not reaching statistical significance; Iranzo et al., 2002), or no change in N2 sleep but an increase in NREM stage 3 (N3; Monaca et al., 2004).

However, quantification of sleep macroarchitectural features may not fully capture the impact of DBS on sleep. In recent years, technical advancements have allowed for a more detailed quantification of neural oscillations during sleep and advanced the field of sleep research (Weiner and Dang-Vu, 2016). Of particular interest are sleep spindles, which are hallmark oscillations of N2 sleep with a frequency of 9–15 Hz that wax and wane in amplitude, lasting 0.5–3 s (Weiner and Dang-Vu, 2016). They are thought to originate from interactions between thalamic reticular, thalamocortical, and cortical pyramidal networks (Weiner and Dang-Vu, 2016). Sleep spindles have been increasingly recognized as critical for declarative memory, sleep-related memory consolidation, as well as sleep maintenance and continuity (Fernandez and Lüthi, 2020).

In contrast to N2 sleep, pertinent electrophysiological features of N3 sleep include both slow waves (SW, <1 Hz), which occur with lower density in PD patients and may be altered in morphology compared to controls (Memon et al., 2023), and power in the delta frequency range (1.0–4.0 Hz). In prior work, we found a reduced SW density in a group of 56 PD patients compared to controls, but no difference in delta spectral power or SW morphological features including peak-to-peak amplitude and slope (Memon et al., 2023). However, other studies have found conflicting results. For example, Brunner and colleagues found that spectral power in the low delta (0.78–1.2 Hz) range was reduced in a group of 9 *de novo* PD patients compared to controls (Brunner et al., 2002). Finally, in addition to individual NREM EEG oscillations, the temporal relationship between SWs and spindles plays a significant role in neural plasticity. SW and spindle phase-amplitude coupling (PAC) promotes memory consolidation, and declines with physiological aging (Helfrich et al., 2018; Muehlroth et al., 2019). In the only prior study investigating SW-spindle PAC in PD, we found higher non-uniformity in SW-spindle coupling in PD patients compared to controls (Memon et al., 2023).

However, to our knowledge, the impact of DBS on sleep microarchitecture is unexplored. We therefore sought to perform a quantitative EEG (qEEG) analysis of polysomnogram (PSG)-derived data to determine the effect of DBS on sleep spindles (density, amplitude, and peak frequencies), SW (density, amplitude, and slope), SW-spindle PAC (coupling angle, strength, and percentage), and N2/N3 spectral power. This *post hoc* analysis utilizes data collected as part of a previously completed clinical trial investigating the impact of STN DBS on objective sleep outcomes in PD (Amara et al., 2017). This study obtained PSG for 3 non-consecutive nights in PD patients treated with STN DBS: one night with DBS OFF, one with conventional HIGH frequency (≥130 Hz) stimulation, and one with LOW frequency (60 Hz) stimulation, finding no significant difference in sleep macroarchitecture due to DBS. For the current analysis, we hypothesized that PSG-derived qEEG with DBS at HIGH frequency would show higher spindle density compared to recordings with LOW frequency DBS and DBS OFF.

## Methods

### Participants

A *post hoc* analysis was conducted on PSG-derived EEG data obtained from a previously completed, within-subject, cross-over study to investigate the impact of STN DBS on objective sleep measures in individuals with PD (Amara et al., 2017). The parent study included 20 individuals with PD who had previously undergone STN DBS treatment (18 unilateral and 2 bilateral STN DBS) at the University of Alabama at Birmingham (UAB) for the management of motor symptoms. At UAB, standard DBS protocol involves the initial placement of a unilateral DBS electrode, with the potential to add a contralateral electrode later if deemed necessary. In all cases, the initial electrode was positioned contralateral to the side of the body most affected by PD. Detailed eligibility criteria have been reported elsewhere (Amara et al., 2017). In summary, eligible participants were those with subjective sleep disturbances, defined as a score > 5 on the Pittsburgh Sleep Quality Index (PSQI; Buysse et al., 1989; Memon et al., 2022) at the time of study entry, and on stable medications and DBS settings, optimized to motor benefit, for at least 6 weeks before enrollment. Exclusion criteria included untreated sleep apnea, narcolepsy, prior brain surgery other than STN DBS, or cognitive impairment that could hinder participation. In the present analysis, additional inclusion criteria required at least 2 of the 3 nights of PSG to have EEG data that was sufficiently free of artifact for the qEEG analysis. Five participants were excluded for not meeting this criterion. Thus, 15 participants were included in the current analysis. Of those 15 participants, 11 participants had PSG for all 3 DBS conditions, 3 participants were missing the ON DBS (≥130 Hz frequency) night, and 1 participant was missing the DBS OFF night, all due to unusable EEG data because of movement or electrical artifacts. The study was registered at clinicaltrials.gov (NCT01769690) and received approval from the UAB Institutional Review Board. All participants gave written informed consent prior to participation.

### Assessments

**Figure 1:**
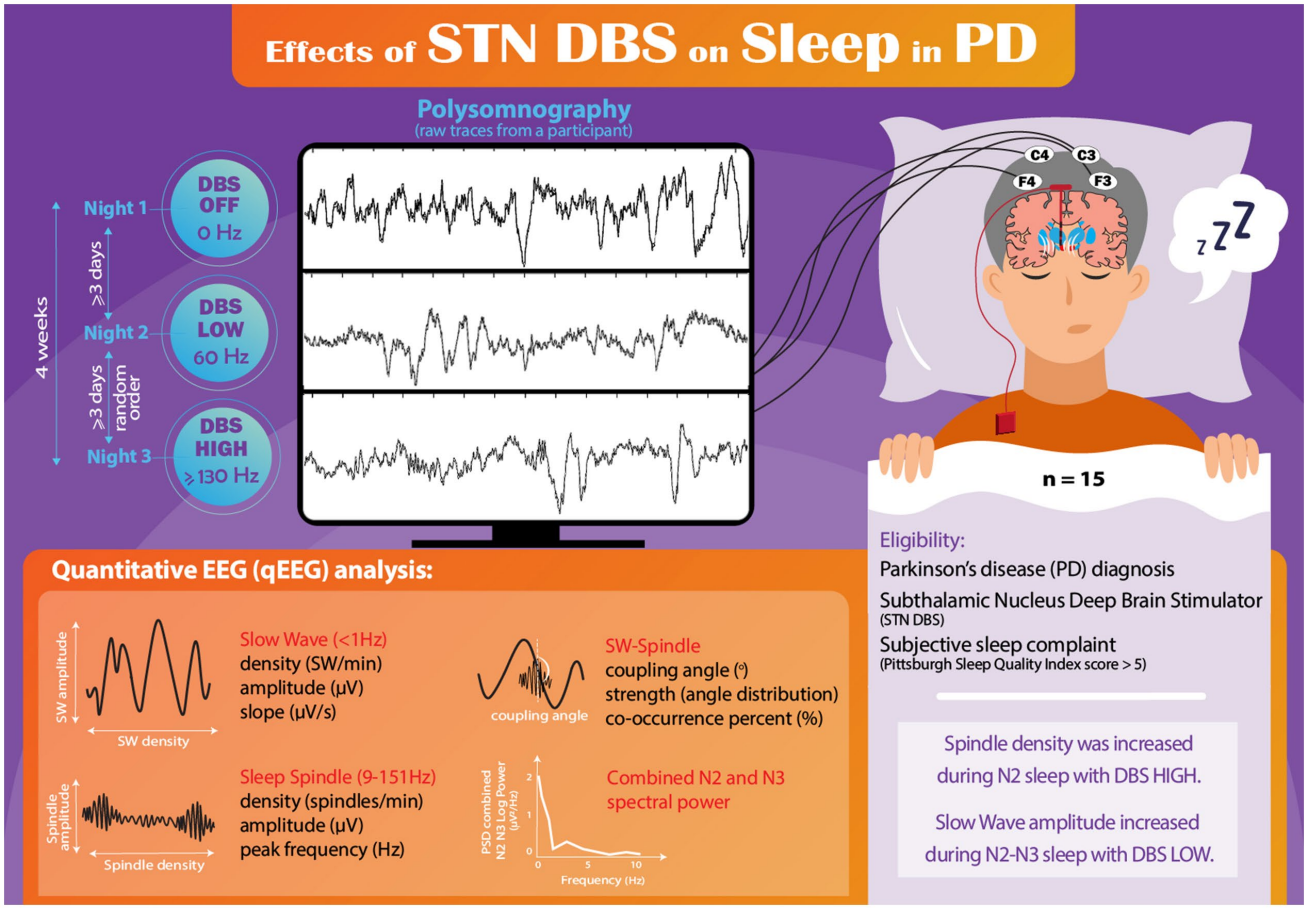
Infographic schematic of the study.

Participants underwent a series of three nonconsecutive nights of PSG. One night involved deactivation of DBS (DBS OFF), another night utilized conventional HIGH-frequency settings (≥130 Hz), and the remaining night employed LOW-frequency settings (60 Hz) using the same amplitude as HIGH frequency condition (Figure 1). Participants adhered to their regular medication regimen throughout the study. The initial PSG study night was with DBS OFF and the order of the HIGH and LOW frequency nights was randomized on the second and third nights, as previously described (Amara et al., 2017). A minimum of three nights separated each PSG session to mitigate any carryover effects. All three PSG studies were completed within a four-week period, and DBS settings were adjusted at 8:00 PM, with the PSG recording beginning at 10 PM. Prior to their initial PSG session, participants were instructed to sleep with DBS OFF for one night at home to ensure their tolerance to sleeping without stimulation. Importantly, no participants withdrew from the study due to intolerance of sleeping with DBS OFF.

Previous research has demonstrated that individuals with monopolar DBS settings experience significant stimulus artifacts during PSG, thereby rendering sleep staging unreliable (Frysinger et al., 2006). Consequently, participants with monopolar configurations (*n* = 7) were systematically reprogrammed to motor-equivalent bipolar settings for the DBS ON and DBS LOW nights, as previously described in detail (Amara et al., 2017). Briefly, bipolar settings were chosen based on participant’s initial DBS programming session monopolar survey and equivalent motor efficacy was determined with the Unified Parkinson’s Disease Rating Scale (UPDRS) part III (Fahn and Elton, 1987) on the DBS OFF night prior to turning DBS off, and again on the 2^nd^ PSG night to confirm efficacy of chosen settings. The same bipolar settings were used on the 2^nd^ and 3^rd^ study nights with the only difference being frequency (≥130 Hz or 60 Hz). Individual participant DBS settings are shown in Supplemental Table 1.

The PSG recordings included EEG obtained from leads F3, F4, C3, C4, O1, and O2, electrooculogram, electromyography of the mentalis, bilateral anterior tibialis, and bilateral extensor digitorum muscles, thermocouple and nasal pressure for airflow monitoring, respiratory effort with chest and abdominal piezoelectric belts, and pulse oximetry. PSG data were independently scored by two certified sleep technicians and a sleep medicine physician (AWA), who were blinded to the DBS settings.

### Quantitative sleep EEG analysis during NREM

#### Preprocessing

All recorded PSG-derived EEG data were converted into European Data Format (EDF) and imported into MATLAB (version R2021b) for subsequent analysis. To identify potential artifacts, each 30-second epoch was visually inspected. The evaluator responsible for assessing the EEG data (AAM) was blinded to DBS settings (raw trace of one representative participant is shown in Figure 1). Comprehensive visual assessment of the F3 and C3 channels was conducted throughout the entire PSG recording, with the identification and removal of any observed electrical or movement-related artifacts. In cases where continuous artifacts persisted in the F3/C3 leads, the F4/C4 channels were utilized for the analysis.

For the DBS OFF nights, 2.3% of N2 and 0.41% of N3 sleep EEG was rejected due to artifact. For the DBS LOW night, the mean artifact-related data rejection rate was 2.8% for N2 and 0.28% for N3. For the DBS HIGH night, 2.6% of N2 and 1.5% of N3 was eliminated due to artifacts.

Due to the spatial and temporal characteristics of SW and spindles (Fernandez and Lüthi, 2020; Timofeev et al., 2020), SW and delta spectral power were examined within frontal leads. Due to the limited number of participants experiencing N3 sleep, especially during the low frequency night (only 4 participants achieved N3 sleep), we analyzed SW and spectral power data from both N2 and N3 sleep stages combined. Sleep spindles, on the other hand, were assessed within central leads, during N2, the predominant stage during which sleep spindles occur. The coupling between SW and sleep spindles was evaluated across stage N2 and N3 using central channels (Helfrich et al., 2018).

### Spectral analysis

The spectral power analysis utilized a Hamming window with a duration of 512 milliseconds and a 50% overlap between consecutive windows, resulting in a frequency resolution of 1 Hz. To determine the absolute spectral power within specific frequency ranges, separate averages were computed for stage N2, N3, and combined N2/N3. The frequency ranges of interest included delta (1–4 Hz), theta (5–8 Hz), alpha (9–12 Hz), and beta (13–30 Hz). Supplementary Figure S1 shows the PSD between three different conditions. Note that the DBS LOW-frequency artifact overlaps with electrical line noise at 60 Hz, but that High DBS artifact is visible at 130 Hz.

### Scalp-SW and sleep spindle event detection

To detect slow waves (SW) and sleep spindles, EEG data without artifacts were processed using a custom MATLAB script that utilized validated algorithms previously employed in studies involving older adults (Mölle et al., 2011; Staresina et al., 2015; Helfrich et al., 2018; Memon et al., 2023).

For SW detection, zero crossings were identified in the F3 channel, unless artifact in F3 prompted use of F4. SW events were defined based on the following criteria: (1) a frequency filter ranging from 0.16 to 1.25 Hz, (2) duration between 0.8 and 2 s, and (3) peak to peak amplitude threshold determined as the 75th percentile of the amplitude across all stage N2, N3 and combined N2 N3 epochs. Artifact-free individual SW events meeting these criteria were then extracted from the raw EEG signal. The following characteristics were computed and averaged across all stage N2 and N3 epochs from the entire PSG recording due to few participants with N3 (*n* = 4 for LOW DBS night): (1) density (number of events per minute), (2) amplitude (peak-to-peak, measured in microvolts), and (3) slope (measured in volts per millisecond).

For sleep spindle event detection, the following parameters were applied in the C3 channel, unless artifact in C3 prompted use of C4: (1) frequency filter ranging from 9 to 15 Hz, (2) amplitude threshold set at the 75th percentile of the amplitude across all stage N2 epochs, and (3) duration range of 0.5 to 3 s. By utilizing Hilbert’s transformation, the analytical amplitude was calculated, and events meeting the specified parameters were automatically extracted. The subsequent sleep spindle characteristics were computed and averaged over all stage N2 epochs from the entire PSG recording: (1) density (number of events per minute), (2) amplitude (peak-to-peak, measured in microvolts), and (3) peak frequency (cycles per second, measured in Hz) for spindles (9–15 Hz), slow spindles (9–11 Hz), and fast spindles (12–15 Hz). The division into slow and fast frequency bins was based on our previous findings that demonstrated a higher slow spindle peak frequency in PD patients compared to non-PD controls (Memon et al., 2023).

### SW locked sleep spindle phase-amplitude coupling

Upon identifying individual SW events, the subsequent step involved determining the instantaneous phase angle of these events by applying the Hilbert transformation to the raw signal. Subsequently, the raw signal was subjected to filtering within the frequency range of 9–15 Hz, corresponding to the spindle frequency range. The Hilbert transformation was employed once again on the filtered signal to obtain the instantaneous amplitude. The maximum amplitude of the spindle and its corresponding phase angle of the SW were then identified (Dvorak and Fenton, 2014; Staresina et al., 2015).

The following characteristics were computed for the combined N2 and N3 sleep stages:

(1) Mean SW phase angle in degrees, which was calculated utilizing the CircStat toolbox (Berens, 2009). This measure provides information regarding the average phase angle of the SW events. (2) Coupling angle distribution nonuniformity or strength, which was assessed using the Rayleigh test statistic. This analysis aids in determining the extent of nonuniformity or clustering in the coupling angles between the phase of SW and the amplitude of the spindle.

### SW-spindle co-occurrence percent:

Using the aforementioned parameters, SW and sleep spindle events were identified as distinct entities. The subsequent step involved quantifying the SW-spindle co-occurrence percentage, which measures the proportion of SW events that coincide with the occurrence of sleep spindles.

To compute the SW-spindle co-occurrence percentage, each SW event was scrutinized to determine if a detected sleep spindle’s center fell within the duration of the SW event. If a sleep spindle was found to co-occur with a specific SW event, it was considered an instance of SW-spindle co-occurrence. The SW-spindle co-occurrence percentage was then calculated by normalizing the number of SW events with co-occurring sleep spindles over the total number of SW events.

### Statistical analysis

This study utilized a within-subject, cross-over design to investigate the difference in sleep qEEG morphological features. Statistical analysis was performed using JMP Pro 16 (SAS Institute, Inc., Cary, NC). For the descriptive statistics, the normality of all variables was assessed using the Shapiro–Wilk test. Differences in qEEG morphological characteristics across the different DBS settings were compared with mixed-model repeated-measures analysis of variance. If significant differences were found between the DBS settings, Tukey’s honesty significant difference (HSD) multiple comparison procedure was performed to determine which settings were different. Given the exploratory nature of our study, we did not apply a correction for multiple comparisons. Instead, we chose to accept the possibility of Type 1 error to avoid rejecting potential associations that could be missed due to Type II errors resulting from correction procedures.

## Results

### Participants characteristics

The demographic characteristics of the participants are provided in Table 1. Table 2 displays the sleep characteristics during the OFF, LOW, and HIGH DBS nights. No significant differences were observed between the DBS settings in terms of objective sleep outcomes for these 15 participants, consistent with the previous report of the full cohort of 20 participants (Amara et al., 2017).

**Table 1:** Baseline demographic and clinical characteristics.

Characteristics	Descriptive Statistics (*n*=)
*N*	15
Age (years)	
Mean ± SD	61.8 ± 9.5
Range	45–77
Sex: *N* (%)	
Male	11 (73.3%)
Female	4 (26.7%)
Duration of disease (DOD; years)	
Mean ± SD	10.0 ± 3.7
Range	5–20
Months since DBS placement	
Median (IQR)	13.5 (8.1–23.4)
Levodopa equivalent dose (LED; mg)	
Mean ± SD	968.9 ± 673.3
Range	0–2247.5
MDS-UPDRS^*^	
OFF DBS, On medication	
Median (IQR)	31.0 (28.8–40.3)
Side of surgery *N* (%)	
Bilateral	1 (6.7%)
Left	9 (60.0%)
Right	5 (33.3%)

Mean ± SD presented for normally distributed data. Median (IQR) reported for non-normally distributed data.

* Performed in the morning following PSG with DBS OFF, medication on.

**Table 2:** Baseline objective sleep characteristics.

Sleep variables	DBS OFF	DBS LOW frequency (60 Hz)	DBS HIGH frequency (≥130 Hz)	*p* value
Sleep efficiency (%)	83 (76–87)	84 (72–89)	86 (73–91)	*F* = 0.46 *p* = 0.635
Total sleep time (min)	381.0 (308.0–400.0)	380.1 (291.0–434.2)	402.5 (342.5–441.6)	*F* = 0.92 *p* = 0.412
Wake after sleep onset (WASO; min)	73.5 (50.1–113.5)	61.6 (39.2–98.5)	44.5 (42.2–89.6)	*F* = 0.66 *p* = 0.525
Sleep latency (min)	8.3 (2.9–14.0)	11.0 (5.5–34.1)	5.5 (3.9–14.0)	*F* = 1.58 *p* = 0.223
N1%	12.0 (7.0–16.0)	13.0 (8.0–21.0)	8.0 (6.0–14.0)	*F* = 1.26 *p* = 0.299
N1 time (min)	46 (26–53.5)	41.5 (23.0–60.5)	30.0 (20.0–42.2)	*F* = 0.25 *p* = 0.784
N2%	71.7 ± 13.1	66.1 ± 20.4	72.2 ± 12.4	*F* = 1.34 *p* = 0.279
N2 time (min)	248.6 ± 66.7	249.5 ± 106.0	274.1 ± 103.1	*F* = 1.07 *p* = 0.356
N3% Range	1.0 (0.0–9.0) 0.0–15.0	0.0 (0.0–0.0) 0.0–19.0	0.0 (0.0–3.0) 0.0–11.0	*F* = 1.77 *p* = 0.189
N3 time (min) Range	3.5 (0.0–37.0) 0.0–60.0	0 (0.0–1.0) 0.0–73.0	0.5 (0.0–311.5) 0.0–28.0	*F* = 2.58 *p* = 0.094
REM %	11.5 ± 9.7	13.9 ± 8.9	12.7 ± 8.5	*F* = 0.74 *p* = 0.487
REM time (min)	42.8 ± 34.4	51.7 ± 35.0	45.2 ± 31.0	*F* = 0.69 *p* = 0.510
Apnea hypopnea index (events per hour)	0.4 (0.0–3.5)	0.3 (0.0–2.2)	0.5 (0.0–2.8)	*F* = 0.59 *p* = 0.563

Mean ± SD presented for normally distributed data. Median (IQR) reported for non-normally distributed data. N1: non-REM stage1; N2: Non-REM stage 2; N3: non-REM stage 3; REM: rapid eye movement sleep.

### Quantitative NREM sleep EEG analysis

#### Sleep spindle characteristics during N2

**Figure 2:**
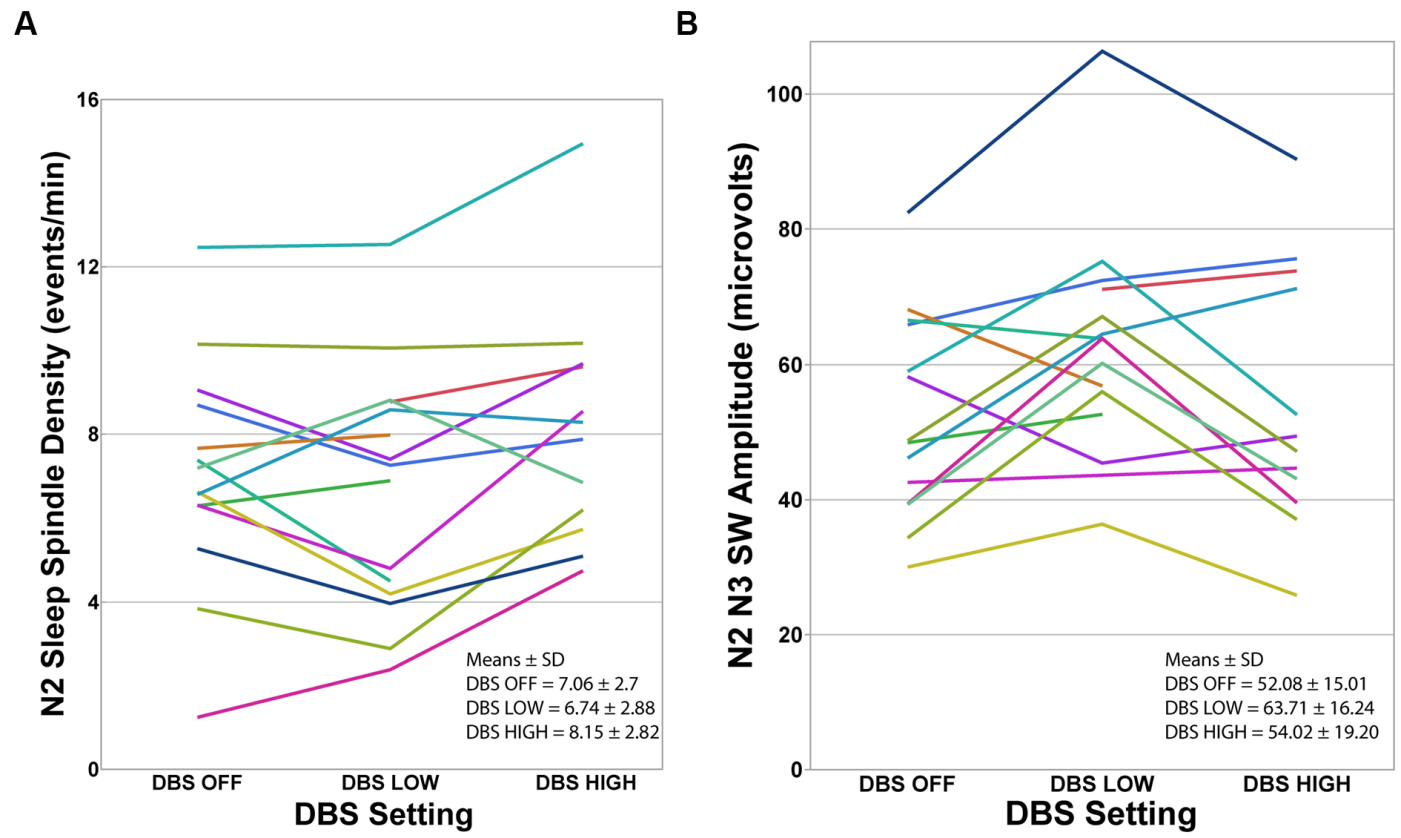
Individual participants at each DBS setting. **(A)** Sleep spindle density during N2 was significantly different between the three DBS conditions using mixed-model repeated-measures analysis of variance (*F* = 5.10, *p* = 0.014). Tukey’s HSD procedure showed that spindle density was higher during DBS HIGH-frequency compared to DBS LOW-frequency. **(B)** Slow Wave amplitude was significantly different between the three DBS conditions using mixed-model repeated-measures analysis of variance (*F* = 5.78, *p* = 0.009). Tukey’s HSD procedure showed that SW amplitude was higher during N2 and N3 in DBS LOW-frequency night compared to DBS HIGH-frequency and DBS OFF. Note that data from one participant during the OFF-night assessment and three participants during the HIGH-night assessment were excluded because EEG was unusable due to movement artifacts.

Sleep spindle density exhibited significant difference between the nights (*F* = 5.10, *p* = 0.014; Figure 2A; Table 3). Tukey’s HSD multiple comparison procedure showed that sleep spindle density was significantly higher on the DBS HIGH night (frequency ≥ 130 Hz) compared to the DBS LOW night. However, no significant differences were observed between the three nights in terms of other spindle morphological features, including peak-to-peak amplitude and peak frequency (Table 3).

**Table 3:** Sleep quantitative electroencephalographic outcomes for each DBS settings.

Sleep qEEG variables	DBS OFF^*^	DBS LOW frequency (60 Hz)^*^	DBS HIGH frequency (≥130 Hz)^**^	*p* value
N2 spindle density	7.06 ± 2.70	6.74 ± 2.88	8.15 ± 2.82	*F* = **5.10** *p* = **0.014**
N2 spindle amplitude	11.82 (10.37–12.90)	12.11 (10.45–14.00)	12.11 (10.31–13.17)	*F* = 0.19 *p* = 0.825
N2 spindle peak-frequency	11.20 ± 0.57	11.16 ± 0.61	11.33 ± 0.55	*F* = 1.43 *p* = 0.260
N2 spindle peak slow frequency	10.25 ± 0.18	10.24 ± 0.22	10.29 ± 0.13	*F* = 0.57 *p* = 0.576
N2 spindle peak fast frequency	13.18 ± 0.09	13.19 ± 0.12	13.24 ± 0.12	*F* = 1.23 *p* = 0.311
N3 slow wave density	11.48 ± 4.25	12.91 ± 3.01	10.11 ± 3.44	*F* = 1.91 *p* = 0.193
N2 N3 slow wave density	3.83 ± 1.07	3.27 ± 1.20	3.53 ± 1.22	*F* = 1.22 *p* = 0.312
N3 slow wave amplitude	52.25 ± 15.00	74.73 ± 2.10	59.80 ± 21.14	*F* = 1.69 *p* = 0.227
N2 N3 slow wave amplitude	52.08 ± 15.06	63.71 ± 16.24	54.20 ± 19.20	***F* = 5.78** ***p* = 0.009**
N3 slow wave slope	91.33 ± 27.25	125.97 ± 17.40	101.50 ± 39.71	*F* = 3.02 *p* = 0.093
N2 N3 slow wave slope	78.20 (63.33–101.13)	98.93 (86.57–127.49)	82.46 (64.50–204.16)	*F* = 1.58 *p* = 0.225
N2 N3 SW-spindle phase amplitude coupling angle	−2.98 ± 1.17	−1.80 ± 1.29	0.52 ± 1.31	F = 1.34 *p* = 0.27
N2 N3 SW-spindle coupling strength	131.88 (41.04–290.13)	143.29 (87.55–381.44)	92.78 (57.81–294.74)	*F* = 1.02 *p* = 0.376
N2 N3 SW-spindle coupling percent	1.39 ± 0.70	1.35 ± 0.91	1.57 ± 1.02	*F* = 0.72 *p* = 0.497
N3 delta power 1–4 Hz	114.08 ± 44.51	170.86 ± 33.71	124.48 ± 66.10	*F* = 1.26 *p* = 0.320
N2 N3 delta power 1–4 Hz	56.89 (35.50–67.84)	65.87 (44.18–70.57)	55.03 (50.11–110.97)	*F* = 1.44 *p* = 0.255
N3 theta 4–8 Hz	10.73 (7.0–11.70)	11.55 (10.36–21.46)	9.11 (6.26–13.83)	*F* = 2.74 *p* = 0.104
N2 N3 theta 4–8	8.25 (5.55–9.77)	8.11 (6.12–10.53)	7.96 (5.84–11.02)	*F* = 1.84 *p* = 0.180
N3 alpha 9–12	1.99 ± 4.51	2.28 ± 4.49	3.90 ± 2.13	*F* = 0.395 *p* = 0.684
N2 N3 alpha 9–12	3.45 (2.66–5.10)	3.32 (2.89–6.43)	3.54 (2.38–4.51)	*F* = 0.454 *p* = 0.640
N3 beta 12–30	0.54 ± 0.24	0.63 ± 0.15	0.52 ± 0.22	*F* = 3.93 *p* = 0.055
N2 N3 beta 12–30	0.68 ± 0.22	0.72 ± 0.26	0.73 ± 0.26	*F* = 1.67 *p* = 0.209

* *N* = 14.

** *N* = 12.Bold values are statistically significant.

### Scalp-SW and spectral power analysis and SW morphology during N2 and N3

To assess the dynamics of sleep EEG activity during stage N2 and N3 under various DBS settings, we investigated the spectral power characteristics. There were no observed differences in the power in any of the measured spectral frequency bands (delta, theta, alpha, or gamma) during N2 and N3 sleep across the three DBS settings. However, SW amplitude was significantly different between the three nights, with Tukey’s HSD procedure showing that SW amplitude was higher on the DBS LOW night compared to DBS OFF and HIGH (Figure 2B; Table 3). There were no statistically significant differences found between the nights in terms of SW morphological characteristics, including density and slope (Table 3).

### SW-spindle phase amplitude coupling characteristics during N2 and N3

There were no significant differences in average phase angle of spindle-SW coupling, the nonuniformity of coupling angles, or the co-occurrence percentage of spindle-SW coupling between PSG nights on different DBS settings (Table 3).

## Discussion

In this *post hoc* analysis of PSG-derived EEG data from a within-subject, crossover study of the effects of HIGH and LOW frequency DBS on objective sleep outcomes, sleep spindle density was higher with DBS on at HIGH frequency than with DBS at LOW frequency. In addition, slow wave amplitude during N2 and N3 sleep was higher with DBS at LOW frequency than with DBS OFF or at HIGH frequency. We did not detect differences between the three conditions in spindle amplitude or peak frequency, SW density or slope, SW-spindle PAC, or spectral power across canonical frequency bands during N2/N3 sleep. Although exploratory, these data may inform future studies investigating stimulation-induced changes in sleep microarchitecture and therefore provide a potential mechanism for intervening on sleep dysfunction in PD with DBS.

STN DBS, although not directly targeted or programmed to address sleep, has been shown to improve both subjective and objective sleep outcomes in PD. Multiple studies have demonstrated improvements in self-reported sleep quality and sleepiness, as measured by the Parkinson’s Disease Sleep Scale (PDSS, PDSS-2; Hjort et al., 2004; Chahine et al., 2011; Nishida et al., 2011; Breen et al., 2015; Deli et al., 2015), PSQI (Iranzo et al., 2002; Monaca et al., 2004; Amara et al., 2012), and Epworth Sleepiness Scale (ESS; Chahine et al., 2011; Baumann-Vogel et al., 2017). In the largest PSG-based study of the effects of DBS on sleep, STN DBS improved total sleep time and sleep efficiency, associated with increased N3 sleep (42.6 ± 34.9 min before DBS, 53.8 ± 43.3 min after DBS) in 50 PD participants (Baumann-Vogel et al., 2017). In another study, total sleep time increased with stimulation on compared to off in 10 PD participants, primarily driven by an increase in N2 sleep (180 ± 23 min with DBS on, 125 ± 16 min with DBS off; Arnulf et al., 2000). Another study of 10 PD patients found that total sleep time increased with DBS on compared to off, but this was due to increases in N3 (69.6 ± 35.4 min with DBS on, 27.7 ± 26.9 min with DBS off) and REM (68.8 ± 35 min with DBS on, 43.1 ± 22.7 min with DBS off; Monaca et al., 2004). There was no significant difference in the time spent in N2 sleep. In the parent study upon which the present analysis is based, there was no difference in total sleep time, sleep efficiency, wakefulness after sleep onset, or time spent in stages N1, N2, N3, or REM between nights with DBS OFF, LOW frequency, or HIGH frequency DBS (Amara et al., 2017). This difference may be related to predominantly unilateral DBS in the current study, compared to bilateral DBS in most other studies. Another possible explanation for this heterogeneity amongst studies is the limitation of qualitative sleep staging. According to the American Academy of Sleep Medicine guidelines, N2 sleep is scored only if K-complexes or sleep spindles are observed during a 30-s epoch (Berry et al., 2018). However, sleep spindles in PD patients are of lower density and have several other morphological differences (including longer duration, slower frequency, and higher maximum peak-to-peak amplitudes) compared to age-matched controls (Christensen et al., 2015; Latreille et al., 2015). This variability in appearance could possibly affect the threshold for spindle detection, and therefore alter the qualitative sleep stage determination.

Nonetheless, DBS does seem to reliably improve at least subjective sleep quality and, in some studies, objective sleep outcomes. Our study provides the first evidence that increased sleep spindle density with high-frequency DBS could be one potential mechanism through which DBS can improve sleep. Spindles play a crucial role in maintaining and sustaining sleep (Astori et al., 2013; Weiner and Dang-Vu, 2016), and as such, their promotion by means of high frequency STN stimulation may underly the improvements in N2 and N3 sleep as well as subjective sleep quality found in the aforementioned studies.

In the only prior study to examine the effect of DBS on sleep spindles, Arnulf et al. recorded sleep spindle density in 6 patients (4 PD, 2 Essential Tremor) during treatment with stimulation of the ventral intermediate nucleus of the thalamus (VIM) and off stimulation (Arnulf et al., 2000). They found no difference in spindle density on versus off stimulation, suggesting that VIM stimulation was not directly affecting spindle generation. The contrasting results of this study with ours may be due to methodological differences, including their inclusion of patients with Essential Tremor. Alternatively, it is possible that these results imply a differential effect of STN versus VIM stimulation on sleep spindles. Although a definite pathophysiological mechanism remains elusive, a growing body of evidence implicates the basal ganglia as an important node in a brain-wide network critical for the maintenance of sleep (Liu and Dan, 2019). In healthy primates, basal ganglia neurons have been shown to exhibit slow oscillations in firing similar to those observed in cortical neurons (Mizrahi-Kliger et al., 2018). Recording of local field potentials (LFPs) in the basal ganglia demonstrate dramatically reduced slow oscillations compared with thalamocortical networks (Mizrahi-Kliger et al., 2018). In the 1-methyl-4-phenyl-1,2,3,6-tetrahydropyridine (MPTP) primate model of parkinsonism, increased power in the alpha and low beta range (10–17 Hz) during NREM was seen in GPe, GPi, and STN (Mizrahi-Kliger et al., 2020). This increase was associated with a decrease in the power of slow oscillatory firing of the basal ganglia, and a decreased propensity for sleep and an increased frequency of awakenings. Furthermore, beta oscillations became more prominent in the approach to awakenings, and in humans, STN LFPs show that beta activity is reduced during NREM (Thompson et al., 2018; van Rheede et al., 2022). Modulation of pathologic synchronized oscillatory activity by high frequency DBS may therefore promote the return of physiological sleep structure, including spindle formation, and thus allow for the maintenance of sleep.

The other significant finding of this study was a difference in SW amplitude in combined N2 and N3 sleep between the three stimulation conditions. Interestingly, SW amplitude was highest with LOW frequency DBS, and lower in HIGH frequency DBS and OFF DBS. The significance of this result is uncertain and must be interpreted with caution. It is important to note that when only N3 sleep was examined, there was no difference in SW amplitude between the three conditions. This may be due to the low number of participants achieving N3 sleep in the DBS low frequency condition (only four subjects). Another possibility is that due to the automated nature of the analysis, K-complexes in N2 sleep may have been interpreted as slow waves and thus caused the discrepancy between N2 and combined N2 and N3 sleep. Still, this finding could motivate future studies of low frequency DBS, perhaps lower than 60 Hz, on SW activity.

Strengths of this study include the utilization of PSG-derived EEG data from a within-subject, crossover study and the use of qEEG analytical methods. An important limitation is the *post hoc* and exploratory nature of the analysis, which is prone to biased interpretation. Nonetheless, such retrospective analyses can provide valuable insights and generate new hypotheses for further exploration and investigation. This study is also limited by the absence of N3 sleep for several participants during the night of low frequency DBS, preventing analysis of N3 sleep for all but four subjects. The exclusion of artifact-contaminated PSG data also may limit our accuracy, though as above the amount of data rejected due to artifact was generally low (2.3–2.8% N2, 0.28–1.5% N3). Furthermore, this was addressed in the parent clinical trial by employing bipolar stimulation configurations in all subjects, which may be less likely to cause stimulation artifact (Frysinger et al., 2006; Amara et al., 2017).

In conclusion, this is the first study to examine the effects of STN DBS on sleep spindle density as well as several other qEEG outcomes during NREM in PD patients. DBS likely has a beneficial therapeutic effect on sleep in PD, which may be due in part to increased sleep spindle density during N2 sleep. Given the exploratory nature of this study, it will be critical for future studies to further examine the potential therapeutic effect of DBS on sleep microarchitecture. These findings have important therapeutic implications and represent a potentially substantial advancement in the search for improved treatments for sleep dysfunction in PD.

## Data availability statement

The raw data supporting the conclusions of this article will be made available by the authors, without undue reservation.

## Ethics statement

The studies involving humans were approved by University of Alabama at Birmingham IRB. The studies were conducted in accordance with the local legislation and institutional requirements. The participants provided their written informed consent to participate in this study.

## Author contributions

AM: Conceptualization, Formal Analysis, Methodology, Visualization, Writing – original draft, Writing – review and editing, Data curation. BE: Data curation, Formal Analysis, Methodology, Writing – review and editing. AB: Writing – original draft, Writing – review and editing. AG: Data curation, Formal Analysis, Methodology, Writing – review and editing. CC: Data curation, Visualization, Writing – review and editing. ZI: Conceptualization, Formal Analysis, Methodology, Supervision, Writing – review and editing. AJ: Data curation, Methodology, Writing – review and editing. SM: Conceptualization, Formal Analysis, Methodology, Supervision, Writing – review and editing. AA: Conceptualization, Data curation, Formal Analysis, Funding acquisition, Investigation, Supervision, Writing – review and editing.

## Funding

The author(s) declare financial support was received for the research, authorship, and/or publication of this article. AA received funding from NIH (K23NS080912 and R01HD100670).

## Conflict of interest

The authors declare that the research was conducted in the absence of any commercial or financial relationships that could be construed as a potential conflict of interest.

## Publisher’s note

All claims expressed in this article are solely those of the authors and do not necessarily represent those of their affiliated organizations, or those of the publisher, the editors and the reviewers. Any product that may be evaluated in this article, or claim that may be made by its manufacturer, is not guaranteed or endorsed by the publisher.
