# Supplementary figures and images for: Effects of deep brain stimulation on quantitative sleep electroencephalogram during non-rapid eye movement in Parkinson’s disease
Source: Front Hum Neurosci. 2023 Sep 21;17:1269864. doi: 10.3389/fnhum.2023.1269864 (PMC10551142; doi:10.3389/fnhum.2023.1269864)

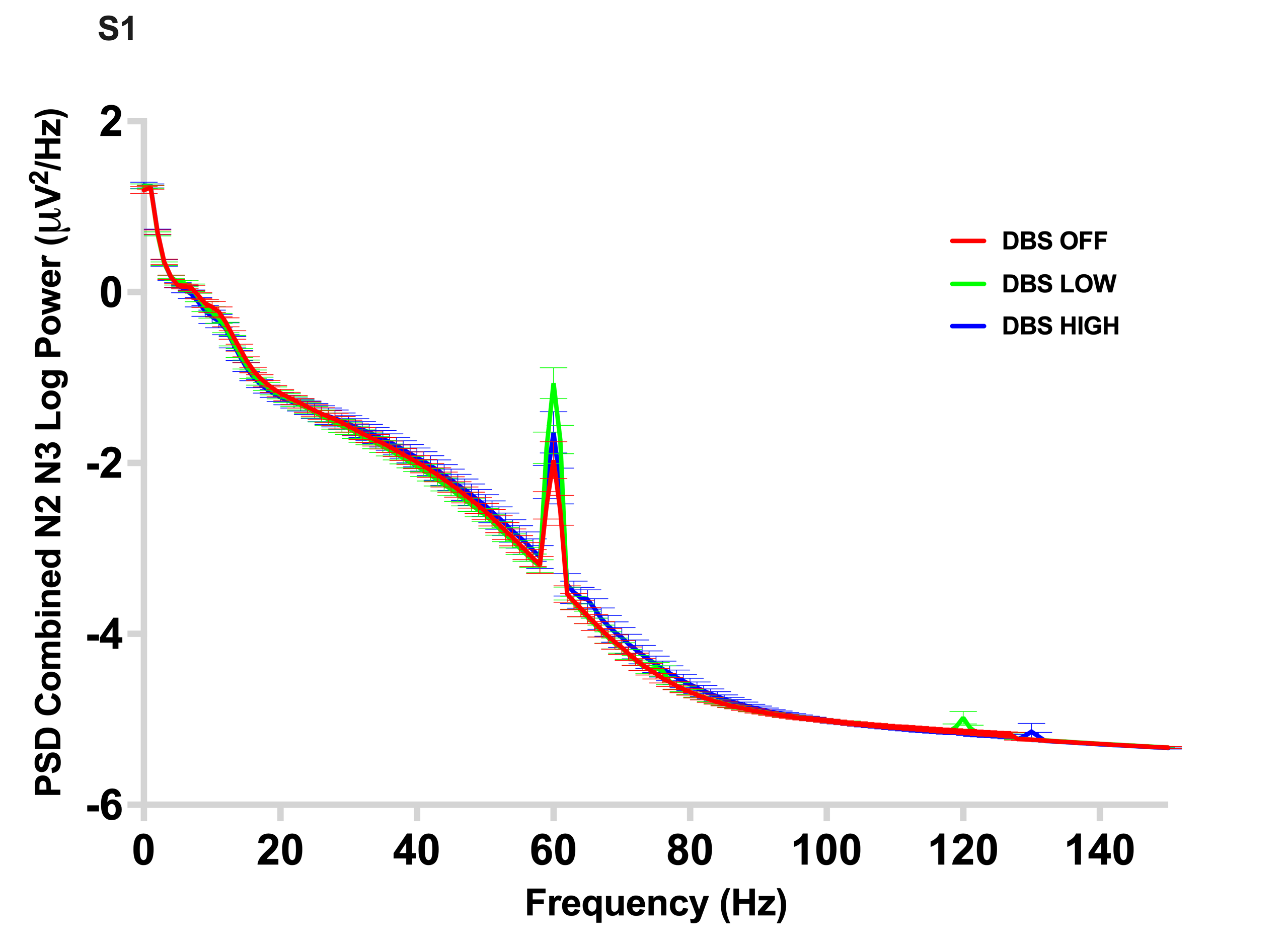

Supplement: Supplementary file 1 [file Image_1.tiff]
