# Supplementary material for: Effects of deep brain stimulation on quantitative sleep electroencephalogram during non-rapid eye movement in Parkinson’s disease
Source: Front Hum Neurosci. 2023 Sep 21;17:1269864. doi: 10.3389/fnhum.2023.1269864 (PMC10551142; doi:10.3389/fnhum.2023.1269864)
Supplement: Supplementary file 2 [file Table_1.docx]

Supplemental Table 1: DBS Settings

| ID |  | Home settings | | | | HIGH frequency DBS Sleep study night | | | |
| --- | --- | --- | --- | --- | --- | --- | --- | --- | --- |
|  | Side of DBS | Contacts | Amp. | Pulse width | Freq. | Contacts | Amp. | Pulse width | Freq. |
| 2 | L | C+ 2- | 3.2 | 90 | 160 | 3+ 2+ | 3.4 | 90 | 160 |
| 3 | R | 1- 3+ | 4.0 | 60 | 160 | Unchanged | | | |
| 4 | L | C+ 1- | 3.8 | 60 | 160 | 3+ 1- | 4.5 | 60 | 160 |
| 5 | R | C+ 2- | 3.3 | 60 | 145 | 3+ 2- | 3.2 | 60 | 145 |
| 10 | R | 1- 3+ | 3.4 | 60 | 160 | Unchanged | | | |
| 11 | L | 0- 3+ | 4.5 | 60 | 160 | Unchanged | | | |
| 13 | L | 2- 3+ | 4.9 | 90 | 180 | Unchanged | | | |
| 14 | R | 2- 3+ | 3.0 | 60 | 130 | Unchanged | | | |
| 15 | L | 2- 3+ | 2.7 | 60 | 160 | Unchanged | | | |
| 16 | R  L | C+ 1-  C+ 2- | 3.5  5.7 | 90  150 | 160  160 | 2+ 1-  3+ 1- | 3.2  4.5 | 90  150 | 160  160 |
| 17 | L | C+ 0- | 5.0 | 100 | 160 | 1+ 0- | 4.8 | 100 | 160 |
| 18 | R | C+ 2- | 4.1 | 90 | 190 | 1- 3+ | 4.0 | 90 | 190 |
| 19 | L | C+ 2- | 4.0 | 90 | 130 | 0- 3+ | 4.3 | 90 | 130 |
| 21 | L | 2- 3+ | 4.2 | 60 | 160 | Unchanged | | | |
| 23 | L | 1- 3+ | 4.0 | 60 | 160 | Unchanged | | | |
